# Seed-associated bacterial community structure and possible transmission to seedling in wasabi, *Eutrema japonicum*

**DOI:** 10.17912/micropub.biology.002222

**Published:** 2026-09-09

**Authors:** Kana Ohata, Sho Hirashima, Yuzuki Akahori, Haruki Kitano, Harunosuke Shoji, Susumu Hisamatsu, Yusuke Katai, Hisae Hirata, Masayoshi Hashimoto

**Affiliations:** 1 Faculty of Agriculture, Shizuoka University, Shizuoka, Japan; 2 Department of Agriculture, Graduate School of Integrated Science and Technology, Shizuoka University, Shizuoka, Japan; 3 National Institute of Technology, Numazu College, Numazu, Japan; 4 Izu Agricultural Research Center, Shizuoka Prefectural Research Institute of Agriculture and Forestry, Izu, Japan

## Abstract

Seeds harbor microbial communities that contribute to enhancing the plant stress resilience. Using 16S rRNA gene profiling, this study characterized the bacterial microbiota of dry and pre-germinated seeds, and three compartments (soil, root, and leaf) from seedlings of wasabi ( *Eutrema japonicum* ). The seed-associated communities exhibited distinct characteristics in terms of the diversity metrics. SourceTracker analysis together with shared amplicon sequence variants (ASVs) between dry and pre-germinated seeds suggests that the seed microbiota may be partially transmitted to the seedling tissues. In particular, Burkholderiaceae among the seed core ASVs dominated the leaf-associated microbiota, suggesting preferential seed-to-leaf transmission in wasabi.

**Figure 1. Bacterial community structure across wasabi seed and seedling compartments and potential microbial transmission from seed to seedling:** **(A)** Photographs of dry seeds (top), pre-germinated seeds (pre seed; middle; scale bar = 1 cm), and a seedling (bottom; scale bar = 3 cm). **(B)** Alpha diversity across five compartments. Different letters indicate statistical significance among compartments based on pairwise Wilcoxon rank-sum test with Benjamini–Hochberg adjustment (adjusted *P* < 0.05). Asterisks indicate statistical significance between samples derived from one seed each and three seeds each in each compartment based on Welch’s t-test ( *P* < 0.05). **(C)** Principal coordinate analysis (PCoA) based on Bray–Curtis distance. Colors indicate compartments. Circles and triangles in seed compartments represent samples consisting of one or three seeds per sample, respectively. **(D)** Taxonomic composition of bacterial communities across compartments at the family level. The 15 most abundant families are shown; remaining taxa are grouped as “others”. **(E)** SourceTracker analysis across compartments. Plant compartments were assigned as sink, and seed and soil compartments as source. Analysis was also performed between seed compartments. **(F)** Euler diagram showing amplicon sequence variant (ASV) overlap between the dry and pre-germinated seed communities. Each area is almost proportional to the number of ASVs it contains. A total of 76 ASVs are shared between the two seed compartments. **(G)** Euler diagrams showing overlap among the 76 seed core ASVs, soil community, and root (top) or leaf (bottom) communities. A total of 15 or 11 ASVs were categorized as root- or leaf-specific seed core ASVs. **(H)** Euler diagram showing overlap between root-specific (n = 15) and leaf-specific (n = 11) seed core ASVs. **(I)** Relative abundance of root- (top) and leaf-specific (bottom) seed core ASVs across the four compartments at the family level. Colors correspond to the legend in panel D.

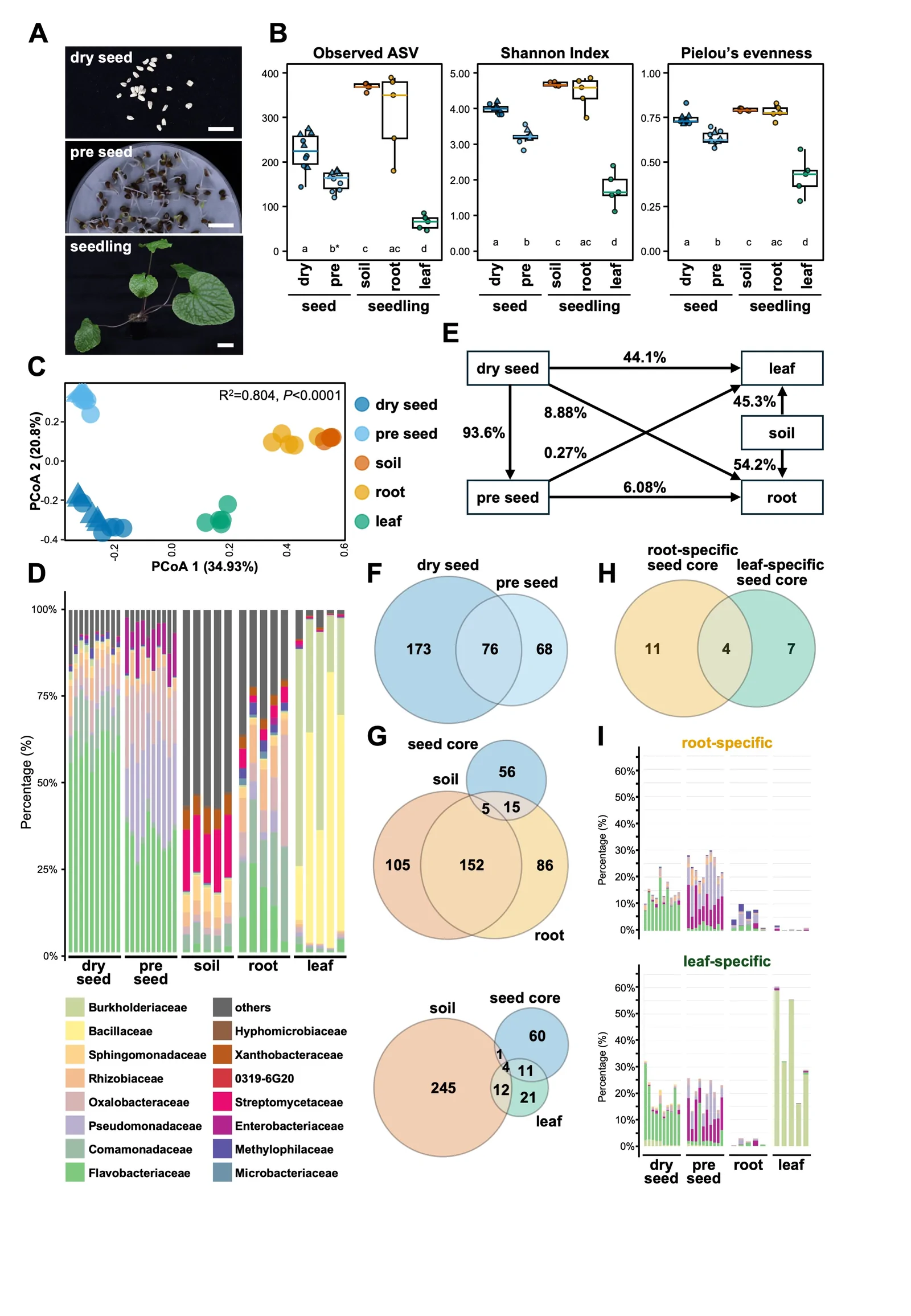



## Description

Seeds harbor distinct bacterial communities that can be transmitted to developing seedlings and subsequent generations, contributing to physiological processes including disease resistance and growth promotion (Shade et al., 2017; Matsumoto et al., 2021; Abdelfattah et al., 2023). A meta-analysis across 50 plant species reported that seeds are inhabited by bacterial communities dominated by Proteobacteria, with core taxa including the genera *Pantoea* , *Pseudomonas* , and *Sphingomonas* shared across many plant species (Simonin et al., 2022). However, seed microbiota dynamics remain uncharacterized in many crops. Wasabi ( *Eutrema japonicum* ) is a Brassicaceae crop originally cultivated in Japan. Traditional wasabi cultivation utilizes unique cultivation fields constructed using natural rocks and sands with water flow on the surface of the field. During the seed collection process, matured siliques were exposed to the water flow alongside the cultivation field for approximately two months to eliminate rotten siliques. This unique agricultural practice in seed collection may contribute to shaping the wasabi seed microbiota. However, to our knowledge, no studies has characterized the wasabi seed-associated bacterial communities or their potential transmission to seedlings. In this study, 16S rRNA gene amplicon sequencing was performed to characterize bacterial communities across five compartments including dry seeds, pre-germinated seeds, and soil, root, and leaf of seedling, all derived from a single seed lot of one variety ( Fig. 1A ).

Bacterial communities in all five compartments were significantly different from each other in both alpha and beta diversity ( Fig. 1B, C). The highest values were commonly found in the soil and root compartments across all alpha diversity indices, while the lowest values in the leaf compartment. Intermediate values were commonly found in the seed compartments. Between the seed compartments, the dry seeds exhibited higher diversity compared with pre-germinated seeds, suggesting pre-germination treatment was associated with a community shift toward higher dominance by a few taxa. In principal coordinate analysis (PCoA) plot, compartment explained 80.4% of the whole variance ( *P* <0.0001). Clear separation was found between the seed and seedling communities according to the first axis (34.93%), and another separation between the dry and pre-germinated seed compartments was also found according to the second axis (20.8%), consistent with the results on other plant species (Kim et al., 2022; Wu et al., 2023).

As shown in Fig. 1D, both seed compartments were dominated by Flavobacteriaceae, a pattern not observed in the seedling compartments. The community shift between dry and pre-germinated seeds was also obvious in family-level relative abundance, with higher relative abundance of Pseudomonadaceae, Oxalobacteraceae, and Enterobacteriaceae, and lower relative abundance of Flavobacteriaceae and Comamonadaceae in pre-germinated seeds.

SourceTracker analysis revealed microbiota transmission across seed and seedling compartments ( Fig. 1E ). The dry seed microbiota was estimated to contribute 93.6% of the pre-germinated seed community. The soil community served as a source of 45.3% and 54.2% of the leaf and root microbiota, respectively. The contributions of both seed compartments to the root community were estimated at approximately 6–9%, while a much higher contribution from dry seeds to the leaf community was observed (44.1%), in contrast to the lower contribution from pre-germinated seeds (0.27%). These results may suggest a higher rate of microbiota transmission from dry seeds to leaves than to roots.

In the Euler diagram ( Fig. 1F ), 76 ASVs were detected in both seed compartments and characterized as seed core ASVs. Of these, 15 and 11 seed core ASVs were also detected in the root and leaf compartments, respectively, and were defined as the root-specific and the leaf-specific seed core ASVs ( Fig. 1G ). Four ASVs were shared between the root-specific and the leaf-specific seed core ASVs ( Fig. 1H ), suggesting the transmission of common ASVs from seeds to root and leaf compartments.

To examine the distribution of both types of seed core ASVs, the relative abundance of each seed core ASV was calculated across the four compartments other than soil ( Fig. 1I ). For the root-specific seed core ASVs, higher abundance was observed in pre-germinated seeds compared with dry seeds. In contrast, the leaf-specific seed core ASVs showed comparable abundances in both seed compartments. In the seedling compartments, the root-specific seed core ASVs accounted for 6.1 ± 3.5% and 0.85 ± 0.73% of the root and leaf communities, respectively. In contrast, the leaf-specific seed core ASVs accounted for 1.8 ± 1.2% and 38.7 ± 18.7% of the root and leaf communities, respectively. In particular, Burkholderiaceae in the leaf-specific seed core ASVs dominated the leaf community (37.8 ± 18.5%), while their contribution was quite limited in the root (~0.01%), dry seed (1.3%), and pre-germinated seed (0.31%) communities. These results suggest that Burkholderiaceae ASVs may adapt to the leaf niche following potential transmission from seed to leaf.

Among the 11 leaf-specific seed core ASVs, three ASVs were assigned to Burkholderiaceae: two to the genus *Burkholderia-Caballeronia-Paraburkholderia* and one to the genus *Ralstonia* . These genera have been reported to be abundant taxa in the seed microbiota of oilseed rape, with their relative abundances differing among three cultivars (Rybakova et al., 2017). Burkholderiaceae has also been identified as a core member of both the root and leaf microbiota in several Brassicaceae plants (Ritpitakphong et al., 2016; Harbort et al., 2020; Karasov et al., 2024; Zhong et al., 2025). However, the relative abundance of Burkholderiaceae in the leaf microbiota has varied considerably among studies, suggesting that the cultivation conditions of the wasabi seedlings including the commercial potting soil and greenhouse environment may have influenced the seedling microbiota. Therefore, further studies are needed to clarify the abundances of these Burkholderiaceae genera in seeds and leaves of different wasabi cultivars under long-term field cultivation.

The most dominant ASV among the three Burkholderiaceae ASVs was closely related to *Paraburkholderia phytofirmans* . *P. phytofirmans* strain PsJN, originally isolated from onion roots, has been reported to promote plant growth in several plant species (Weilharter et al., 2011; Poupin et al., 2013). In contrast, the ASV classified as *Ralstonia* is a potential soil-borne pathogen. The present study does not provide functional evidence regarding the roles of these Burkholderiaceae ASVs on wasabi leaves. Thus, it is important to isolate and functionally characterize these bacterial strains from wasabi seeds and leaves.

## Methods

**Plant materials and sample harvesting**

All plant materials were derived from a single seed lot of wasabi cultivar Izuma, and prepared at the Izu Agricultural Research Center, Shizuoka Prefectural Research Institute of Agriculture and Forestry. Matured siliques were collected from parental plants raised in a greenhouse and immersed in a natural water stream for two months in a canal of the wasabi research field managed in the *tatami-ishi* -style system. This process removed rotten siliques by water stream, resulting in only matured seeds. The collected seeds were air-dried at 5–10˚C until 30% of their initial fresh weight for one week, and stored at 0˚C until further use. To break seed dormancy, dry seeds were treated with 100 ppm gibberellin (Meiji Seika Pharma, Japan) at 5˚C for 11 days, rinsed with distilled water, incubated under moist conditions at 5˚C for 8 days, and then at 15˚C for 2 days to obtain pre-germinated seeds with radicles. Seedlings were raised to the 4–5 expanded leaf stage in cell trays filled with commercial potting soil (Tanemaki Baido, Takii, Japan) in a greenhouse at ambient temperature for approximately 3 months.

All samples were harvested on the same day in October 2022. For soil, root, and leaf compartments, five independent biological replicates were obtained from individual seedlings as described previously (Hashimoto et al. 2026). For seed compartments, dry or pre-germinated seeds were transferred into Lysing Matrix E tubes (MP Biomedicals, CA, USA) without surface sterilization using sterilized forceps to reflect real cultivation conditions. To capture possible seed-to-seed variation in each seed type, five biological replicates with one seed each and another five replicates with three seeds each were prepared.

**16S rRNA gene sequencing**

Total DNA was extracted using FastDNA SPIN Kit for Soil (MP Biomedicals) as described previously (Hashimoto et al. 2026). DNA concentrations were quantified using Quant-iT PicoGreen dsDNA Assay Kit (Thermo Fisher Scientific, MA, USA) on a LightCycler 480 system (Roche, Basel, Switzerland). Ten nanograms of total DNA was added to a 10 μL first PCR reaction mix containing KAPA HiFi HotStart ReadyMix (KAPA Biosystems, MA, USA). The V5–V7 region of the bacterial 16S rRNA gene was amplified using primers 799F-Nex (5′-TCGTCGGCAGCGTCAGATGTGTATAAGAGACAG-NNN-AACMGGATTAGATACCCKG-3′) and 1192R-Nex (5′-GTCTCGTGGGCTCGGAGATGTGTATAAGAGACAG-NNN-ACGTCATCCCCACCTTCC-3′) at a final concentration of 300 nM, with 35 cycles of denaturation at 94˚C, annealing at 58 ˚C, and extension at 72 ˚C for 30 s each.

The N region in each primer consists of three to six random nucleotides incorporated at equimolar ratios to increase library diversity (Lundberg et al., 2013). Five microliters of first PCR products were treated with 2 μL ExoSAP-IT reagent (ThermoFisher Scientific, MA, USA) to remove residual primers and dNTPs. Two microliters of the products were added to a 20 μL second PCR reaction mix to obtain dual-barcoded PCR products. A second PCR of ten cycles was performed under the same temperature conditions as the first PCR. Primers were designed according to Toju et al., 2019. Bacteria-derived PCR products were purified using QIAquick Gel Extraction Kit (QIAGEN, Venlo, Netherland). After measuring DNA concentration using PicoGreen, PCR products were pooled and purified twice using KAPA HyperPure Beads (KAPA Biosystems) at a 1:0.8 volume ratio. Paired-end sequencing (2 × 250 bp) was performed on the Illumina NovaSeq 6000 platform at Novogene (Beijing, China). Adapter sequences and poly-G tails were removed from raw reads using fastp. Primers were trimmed, and reads were quality-filtered, denoised, and merged into amplicon sequence variants (ASVs) using the cutadapt and DADA2 plugins within QIIME2 version 2021.8 (Bolyen et al., 2019). Taxonomic classification was performed using the SILVA database (version 138).

**Bioinformatic analyses**

Downstream analyses including data visualization and statistical tests were performed in RStudio (v2026.01.0+392), as described previously with slight modifications (Hashimoto et al. 2026). ASVs assigned to mitochondria, chloroplasts, and Archaea, and those with a relative abundance of less than 0.05%, were removed. The remaining ASVs were rarefied to sequencing depths of 10,314, 55,123, 8,528, and 3,733 sequences per sample for seed, soil, root, and leaf compartments, respectively, using the rrarefy function in the vegan package to minimize sequencing depth bias. Analysis of seed core ASVs was performed using ASVs with a relative abundance of more than 0.05% in at least two samples in each compartment. Euler diagrams were generated using the eulerr package.

## Reagents

**Table:** 

**Primer Name**	**Sequence**	**Purpose**
799F-Nex	TCGTCGGCAGCGTCAGATGTGTATAAGAGACAGNNNAACMGGATTAGATACCCKG	1st PCR
1192R-Nex	GTCTCGTGGGCTCGGAGATGTGTATAAGAGACAGNNNACGTCATCCCCACCTTCC	1st PCR
fusion-F-X	AATGATACGGCGACCACCGAGATCTACACXXXXXXXXTCGTCGGCAGCGTC	2nd PCR
fusion-R-X	CAAGCAGAAGACGGCATACGAGATXXXXXXXXGTCTCGTGGGCTCGG	2nd PCR
